# The Use of Fruit and Vegetable by-Products as Enhancers of Health Status of Piglets after Weaning: The Role of Bioactive Compounds from Apple and Carrot Industrial Wastes

**DOI:** 10.3390/vetsci11010015

**Published:** 2023-12-28

**Authors:** Gina Cecilia Pistol, Ana-Maria Pertea, Ionelia Taranu

**Affiliations:** Laboratory of Animal Biology, INCDBNA-IBNA, National Research—Development Institute for Animal Biology and Nutrition, 077015 Balotesti, Ilfov, Romania; ana.pertea@ibna.ro (A.-M.P.); ionelia.taranu@ibna.ro (I.T.)

**Keywords:** weaning piglets, apple by-products, carrot by-products, feed, bioactive compounds, intestinal health

## Abstract

**Simple Summary:**

The ban of antibiotics as growth promoters and zinc oxide (ZnO) as medicinal to reduce diarrhea in weaning piglets, and the use of alternative nutritional modulators during weaning stress are under debate and intensively studied. Of these, the dietary inclusion of by-products rich in bioactive compounds could be a promising weaning-induced disturbance in piglets. This review describes nutritional studies which investigated the effects of bioactive compounds derived from fruit (apple) and vegetables (carrot) or their by-products on the intestinal architecture and function, inflammatory processes and oxidative stress at the intestinal level. Data on the associated signaling pathways and on the microbiota modulation by bioactive compounds from these by-products are also presented.

**Abstract:**

At weaning, piglets are exposed to a large variety of stressors, from environmental/behavioral factors to nutritional stress. Weaning transition affects the gastrointestinal tract especially, resulting in specific disturbances at the level of intestinal morphology, barrier function and integrity, mucosal immunity and gut microbiota. All these alterations are associated with intestinal inflammation, oxidative stress and perturbation of intracellular signaling pathways. The nutritional management of the weaning period aims to achieve the reinforcement of intestinal integrity and functioning to positively modulate the intestinal immunity and that of the gut microbiota and to enhance the health status of piglets. That is why the current research is focused on the raw materials rich in phytochemicals which could positively modulate animal health. The composition analysis of fruit, vegetable and their by-products showed that identified phytochemicals could act as bioactive compounds, which can be used as modulators of weaning-induced disturbances in piglets. This review describes nutritional studies which investigated the effects of bioactive compounds derived from fruit (apple) and vegetables (carrot) or their by-products on the intestinal architecture and function, inflammatory processes and oxidative stress at the intestinal level. Data on the associated signaling pathways and on the microbiota modulation by bioactive compounds from these by-products are also presented.

## 1. Introduction

The agro-food industry generates, after the processing of the raw fruits and vegetables, a large amount of waste (peels, skin, stems, leaves, seeds, roots, etc.). This waste encompasses one third of the food dedicated to human consumption [1], causing environmental issues [2].

Therefore, the solution to this problem could be its use in animal nutrition to improve the animals’ performances and general health status [3], reducing pollution and garbage accumulation, and encouraging the concepts of sustainable diets and circular economy [4]. These by-products of the plant are rich in fiber [5] a as well as other compounds like minerals, vitamins [6], and phenolic compounds [7].

From 2006, according to the European Parliament and Council Regulation EC No. 1831/2003, the European Commission (EC) banned the use of in-feed antibiotics as growth promoters in animal feed [8]. Also, further restrictions on the use of pharmaceutical levels of zinc oxide (ZnO) in piglet diets were implemented from June 2022 [9,10]. Hao et al. reported, however, that the ban of in-feed use of antibiotics has detrimental impacts on animal health and welfare in EU, resulting in an increase in the rate of infections in animals and a decrease in animal production being registered [11]. In post-weaning piglets, an increased emergence of *E. coli* and *Lawsonia intracellularis* infection was detected, and, consequently, a considerable increasement of the rate of morbidity and of mortality was observed [11,12]. Nevertheless, the ban of antibiotics in animal production systems led to a reduction in antibiotic resistance, the reduction in the prevalence of antibiotic-resistant bacteria (*Enterococci*, *Campylobacter*, *Salmonella* species) being registered [11].

The ban of pharmaceutical ZnO (in high concentration) because of the environmental problems it can create has increased the need to find new strategies and solutions for the reduction in post-weaning diarrhea (PWD), mortality, and morbidity, important goals to be achieved [10].

As result, a wide range of nutritional studies aimed to promote gut health in piglets after weaning have been conducted, with a special focus on new sources with high content of bioactive compounds. Of these, fruit and vegetable by-products are rich in functional ingredients with well-demonstrated health benefits, such as antioxidant, anti-inflammatory, antimicrobial and anticarcinogenic effects [13]. The composition analysis of both fruit and vegetable by-products showed that, apart from dietary fiber, these by-products are rich in polyphenolics, carotenoids, tocopherols, vitamin C, and others [14]; the bioactive compounds identified in these by-products could be used as modulators of weaning-induced disturbances in piglets.

This review describes the use of fruit (apple) and vegetables (carrot) and their by-products in the nutrition of post-weaning piglets. The effects of bioactive compounds identified in these by-products on the intestinal architecture and function, inflammatory processes, oxidative stress and autophagy at the intestinal level are taken into account. Data on the associated signaling pathways and on the microbiota modulation by bioactive compounds from these by-products are also presented.

The literature data used for this review paper were retrieved from PubMed^®^ and Google Scholar and the following keywords were used: composition in bioactive compounds of apple wastes; composition in bioactive compounds of carrot wastes; intestinal disturbances in piglets after weaning; apple wastes/apple bioactive compounds and weaning piglets; carrot wastes/carrot bioactive compounds and weaning piglets; apple wastes/apple bioactive compounds and intestinal morphology; apple wastes/apple bioactive compounds and intestinal inflammation; apple wastes/apple bioactive compounds and intestinal oxidative stress; apple wastes/apple bioactive compounds and intestinal signaling pathways; apple wastes/apple bioactive compounds and microbiota; carrot wastes/carrot bioactive compounds and intestinal morphology; carrot wastes/carrot bioactive compounds and intestinal inflammation; carrot wastes and intestinal oxidative stress; carrot wastes/carrot bioactive compounds and intestinal signaling pathways; carrot wastes/carrot bioactive compounds and microbiota. The following keywords were searched on Feedipedia, an on-line encyclopedia of animal feeds website (https://www.feedipedia.org/, accessed on 31 August 2023) and on PubMed^®^ and Google Scholar (accessed on 31 August 2023) apple distribution and chemical composition; carrot distribution and chemical composition. Two hundred fifteen (215) articles were included in this review. The literature was summarized according to the main topics of this review, namely the composition of apple and carrot wastes, weaning disturbances, the effects of apple and carrot wastes on intestinal health in weaning piglets, and the effects of apple and carrot wastes on microbiota in weaning piglets. This review provides valuable insights into the potential of apple and carrot and their by-products rich in bioactive compounds in alleviating weaning-induced disturbances in piglets.

## 2. Weaning Transition in Piglets and the in-Depth Mechanisms of Intestinal Disturbances

In the intensive swine production systems, piglets are usually weaned very early, between 2 and 5 weeks of age. After weaning, piglets must adapt to a solid diet, less digestible and palatable than the sow’s milk [15]. Along with this nutritional stress, weaning piglets are exposed to other abrupt and simultaneously social, environmental and behavioral stressors. In addition to all these factors, an undeveloped enzymatic and immune system leads to a reduced feed intake, sub-optimal or negative growth rates, and increased susceptibility to pathogen infections [15,16]. The gastrointestinal tract is strongly affected by weaning, and the dietary changes result in an insufficient utilization of nutrients, local inflammation and oxidative stress [17,18,19], resulting in a high incidence of diarrhea and, in severe cases, increased morbidity and mortality [20]. Research from the last decade showed that the gastrointestinal tract’s roles are not limited to nutrient digestion and absorption, but also have secretory (mucins, immunoglobulins) and important immune functions, with the intestine being considered the largest immune organ of the body [21,22]. These functions of the gastrointestinal tract are influenced by the interplay between its architectural integrity, nutrition, mucosal barrier and microbiota [23].

The gastrointestinal disturbances that appeared immediately after weaning resulted from changes in both the structure and functionality of the intestine. The weaning transition leads to profound changes in the histological features of the small intestine, affecting mainly the digestion and absorption processes [24,25]. For example, decreased villus height and increased crypt depth were observed in all segments of the small intestine [24,25,26,27,28]. Also, alterations in the intestinal permeability and a higher secretory activity were found in both the small intestine (jejunum) and in the colon of weaned piglets [29,30]. The weaning transition led to the dysregulation of the first line of defense against the pathogens, consisting of a layer of epithelial cells sealed by tight-junction protein complexes, such as claudins (CLDN), occludins and zonula occludens (ZO) proteins [31,32]. For example, a decreased expression of occludin, *CLDN-1*, *ZO-1*, *ZO-2*, and *ZO-3* proteins and genes was detected in the jejunum and ileum of piglets [32,33,34,35]. This damage of the intestinal barrier is also due to a transient intestinal inflammation [36], the intestinal mucosa releasing pro-inflammatory mediators (e.g., interleukins—ILs—and tumor necrosis factor-α—TNF-α) [24]. In weaned piglets, an upregulation of TNF-α and interleukins (IL-1β, IL-6, IL-8, IL-10, IL-12α, IL-22) pro-inflammatory cytokines in the jejunum [37,38], ileum [39] and colon was registered [40]. Also, a reduction in immunoglobulin A (IgA) in feces was reported in weaning piglets [41]. Intestinal inflammation is directly related to and maintained by an excessive generation of reactive oxygen species (ROS), leading to oxidative stress [42,43]. In weaned piglets, the levels of pro-oxidant makers (malondialdehyde MDA, protein carbonyl, DNA oxidation) were significantly increased in the intestine, liver, and plasma [44,45,46,47,48,49], while the markers of antioxidant response (glutathione peroxidase (GSH-Px) and superoxide dismutase (SOD)) were reduced [27,50,51].

Current studies have confirmed that the weaning-induced intestinal disturbances in piglets are modulated by signaling pathways’ nuclear factor kappa-light-chain-enhancer of activated B cells (NF-κB) [52], mitogen-activated protein kinases (MAPKs) [53,54] and Kelch-like epichlorohydrin-associated protein 1 (Keap1)/nuclear factor erythroid 2–related factor 2 (Nrf2) [46,55,56]. The weaning transition induced the upregulation of p38, c-Jun N-terminal kinases (JNKs) and extracellular signal-regulated kinases (ERK) MAPKs [32] and the activation of the NF-κB in the jejunum [38], leading to the over-secretion of pro-inflammatory cytokines, maintaining the intestinal inflammation in piglets after weaning [57]. Also, a cross-talk between oxidative stress and inflammation via NF-κB signaling was already demonstrated [58]. The counterpart of NF-κB, Nrf2 transcription factor, was also affected by the weaning transition in piglets. At weaning, a decreased expression of jejunum *Nrf2* and of its substrate *NQO1* genes was found [47,59].

Recent studies demonstrated that weaning is frequently associated with microbiota dysbiosis [60], with the alterations in gut microbiota composition being one of the major causes for post-weaning diarrhea. Recent studies have demonstrated that, in pigs, the most dominant phyla were *Bacteroidetes*, *Firmicutes*, *Proteobacteria* and *Fusobacteria* [61,62,63,64,65]. Post-weaning diarrhea was associated with an increased relative abundance of *Prevotella*, *Campylobacter*, *Sutterella*, *Roseburia*, *Coprococcus*, *Dorea* and *Lachnospira* and *Fusobacteriaceae* [24,62,66], while the relative abundances of *Bacteroidaceae* and *Enterobacteriaceae*, *Bifidobacterium*, *Bacillus*, *Lactobacillus* and *Ruminococcus* declined over the weaning period [60,66,67,68]. Li et al. [61] observed a reduction in *Alloprevotella* and *Oscillospira* bacteria population in weaned piglets [61], with both species having positive effects on the gut barrier and anti-inflammatory roles [69,70], not only regarding bacteria distribution, but also the functional capacities of the microbial community, in terms of short-chain fatty acids’ (SCFAs) production [71]. In weaned piglets, the levels of microbial metabolites butyrate and acetate were increased [72] due to an increase in the abundances of SCFAs producing bacteria, including *Faecalibacterium*, *Oscillibacter*, *Roseburia*, and *Prevotella* [73,74].

The nutritional management of the weaning period aims to achieve improvements in nutrient digestion and absorption. Also, the reinforcement of intestinal integrity and functioning, and the positive modulation of intestinal immunity and of the gut microbiota led to the enhancement of the health status of piglets and to the attenuation of the weaning stress effects [75,76]. That is why the investigation of new sources of bioactive compounds able to accomplish these important points of a nutritional strategy is necessary.

## 3. The Basal Composition and Bioactive Compounds of Apple and Carrot by-Products

### 3.1. The Proximate Chemical Composition of Apple and Carrot Pomace

Apple (*Malus domestica*) is in fourth place regarding the global consumption of fruits [77], being widely cultivated, especially in the temperate regions [78]. According to FAOSTAT (2022) [79], the global crop production of apples has increased by 48% over the last two decades, reaching 93.14 million tons, with 21.6 million tons of residues (pomace, peels, skin, seeds and stems). Apple pomace is a waste from the processing of apple juice and cider, mainly consisting of skin, seeds, and stems [80]. Due to its composition in carbohydrate, pectin, crude fiber and minerals, it is used in animal feeding [81]. The chemical composition of apple pomace is very variable due to the variety/cultivar of apples, maturity and season of harvest, and due to large variations in the proportion of apple residues in the by-product. Apple pomace is generally poor in protein (3–11% of dry matter, DM) and rich in fiber (11.6–44.5%) and sugars (54.4%) (Table 1). The fibers identified in apple pomace are cellulose (12.0–23.2%), lignin (6.4–19.0%), pectin (3.5–18%) and hemicellulose (5.0–6.2%) (Table 1) [77]. The types of sugar vary as follows: high in fructose (14 to 35% of DM); saccharose and glucose contents are lower (1-11% and 6-13% of DM, respectively) [82,83]. Also, some minerals, such as P (0.07–0.076%), Ca (0.06–0.1%), Mg (0.02–0.36%) and Fe (31.8–38.3%) were found in apple pomace [77,80,84] (Table 1).

Carrot (*Daucus carota* L.) is an annual or biennial herb with a long tradition of use as feed to livestock [95]. The use of carrots in animal feeding has a decreased tendency [95], with carrots being used as feed only during periods of overproduction [96]. Occasionally, other carrot products including the tops resulting from harvesting, and various by-products of carrot processing are fed to livestock [97]. After the extraction of juice, about a third of the raw material is collected as pomace [98]. Similar to apple, carrot pomace has a relatively low protein content (6.21–12.87% of DM) and a high amount of fiber (8.8–28% DM) [99,100] (Table 2). It is a rich source of total sugars (64.3%), mostly sucrose, and also contains macro- and micro-minerals [101]. The lipid content of carrot residues was estimated to be between 1.23 and 2.72% of DM (Table 2) [99,100]. The ash content was increased (6.18–7.67%), indicating high mineral content [99,102], with the carrot waste being a good source of calcium, phosphorus and having a high iron content (11.6–22.3%) [89,99] (Table 2).

### 3.2. The Composition in Bioactive Compounds of Apple and Carrot Pomace

Apart from their composition in proteins, fiber and sugars, these by-products have a broad content of active compounds with potential benefits for health. Apple and apple wastes are rich in fiber, with an average content of 43.6% in apple pomace [93]. Fiber from apple and especially pectin, which is the main soluble dietary fiber found in apple wastes (3.5–18% of dry weight) (Table 1) [90], have anti-inflammatory properties, promoting the gut health [105]. Besides components with a high concentration in apple and apple wastes, carbohydrates are valuable compounds with health benefits. In apples, carbohydrates represent 14% of the nutrient composition [90]. In apple pomace, the simple carbohydrate (mono- and disaccharides) content was greater than in apple itself, being reported as 2.5–12.4% (glucose), 14–35% (fructose) and 3.4–24% (sucrose) [82,83,85,93], with all these being easily absorbed in the small intestine and used as an immediate source of energy [106]. The unabsorbed carbohydrates can pass through the small intestine and positively manipulate the commensal bacteria, which, in turn, help to reduce intestinal inflammation [107]. Also, apple pomace contains vitamins E and C (22.4 and 5.5 mg/100 g, respectively) [90,93] (Table 3). This high content of vitamins E and C, which have been demonstrated to be potent scavengers of free radicals, was associated with the antioxidant properties of apple wastes [90].

It was demonstrated that the content of bioactive compounds in apple pomace is higher than in fresh fruits [111]. This is the case for polyphenols, which are higher in content in pomace, being mainly located in skin (95%) [93]. Polyphenols are among the main valuable constituents of apple pomace due to their well-demonstrated anti-inflammatory, antioxidant and antibacterial properties [112]. During digestion, polyphenols can interact with other molecules like proteins, digestible carbohydrates, and dietary fiber (e.g., pectin), which protect phenolic compounds from oxidation and degradation during gastrointestinal digestion [113,114]. A part of polyphenols is released from their matrix and becomes bioaccessible along the intestinal tract to accomplish its physiological functions, protecting from inflammatory and oxidative-stress-related diseases [113,115,116,117]. Because these processes are essential in piglets weaning phase, many studies presented in this review focused on the effects produced by polyphenols from apple and apple by-products. The total polyphenol content of apple pomace is variable, being reported between 159 and 1062 mg gallic acid equivalents (GAE)/100 g of DM total polyphenols, due to the apple varieties, harvest year and the extraction method used [109,118,119] (Table 3). The anthocyanin content of apple pomace was reported to be similar to that found in whole apple, 2.11% [89]. Flavan-3-ols (catechins, procyanidins) and flavonols (quercetin and its glycosides) were identified as major phenolic compounds in apple pomace [120]. Among flavan-3-ols, the catechin content of apple pomace was reported to be between 0.94 and 12.7 mg/100 g DM [109,121,122]; the content of procyanidin B2 was 2.61–16 mg/100 g DM [109,121,122,123]; and the epicatechin levels have been reported between 0.76 and 19 mg/100 g DM [109,124]. Also, phenolic acids (represented mainly by chlorogenic acid) are found in substantial amounts in apple pomace [109]. The level of chlorogenic acid has been reported to be between 1.43 and 41.55 mg/100 g DM [109,123,124,125]. Although the composition of polyphenols in apple pomace was intensely studied and reported, new phenolic compounds have been isolated and identified. For example, Ramirez-Ambrosi et al. [126] identified 52 phenolic compounds using UHPLC-DAD–ESI-Q-ToF-MS (ultra-high-performance liquid chromatography with diode array detection coupled to electrospray ionization and quadrupole time-of-flight mass spectrometry). In this study, three flavonols (isorhamnetin-3-O-rutinoside, isorhamnetin-3-O-pentosides and isorhamnetin-3-O-arabinofuranoside) were identified for the first time in apple pomace. Another compound identified in recent years in apple pomace, monoterpene–pinnatifidanoside D, exhibited small antiplatelet aggregation activity [127]. Mohammed and Mustafa [128] and Khalil and Mustafa [129] isolated novel furanocoumarins from apple seeds, compounds which exhibited promising antimicrobial activity against Escherichia coli, Pseudomonas aeruginosa, Candida albicans, and Aspergillus niger.

Carrot is a rich source of vitamin C and contains about 300–700 mg/kg DM of this vitamin [95] (Table 3). Also, carrot pomace has a high content of digestible carbohydrates of 20–50 g/100 g dry weight [102,130]. An important benefit of carrot wastes is their high carotenoid content, and especially ß-carotene, which is a precursor of vitamin A (retinol) involved in eye function, reproduction, growth and the integrity of skin and mucous tissues. The content of carotene depends on the carrot variety [131]; orange carrots contain 200–1000 mg/kg (DM) of ß-carotene [95], while in a study of Shyamala et al. [101], the total carotene and ß-carotene content of carrot pulp waste was 4.0 and 3.92 mg/100 g, respectively (Table 3).

The total phenolic content of the whole carrots is lower than in apple, ranging from 159 to 259 mg/kg depending on the carrot variety [132], but in the peels, it is almost 13.8 g/kg [133] (Table 3). In carrot pomace, chlorogenic acid accounted for more than 82% of the total content of phenolic acids. Furthermore, the dihydroxybenzoic acid protocatechuic acid was detected in high amounts in carrot (10.9 mg/kg) in comparison to other phenolic acids [102,133,134]. Also, high levels of tannins were recorded in carrot wastes (318 mg/100 g) [102].

### 3.3. The Bioaccessibility of Phenolic Compounds

The absorption of major phenolics occurs mainly in the small intestine [135], with the intestinal epitheliums being the main physical and biological barriers involved in the absorption of nutrients [136]. After gastrointestinal digestion, phenolic compounds follow two different behaviors: the intestinal bioaccessible fraction, potentially absorbed in the small intestine, and the insoluble non-bioaccessible fraction, which had occurred in vitro colonic digestion. There are studies showing that different phenolic compounds showed different rates of absorption in the small intestine [137]. Liu et al. [138] found that partial degradation of chlorogenic acids occurred under weak alkaline conditions. A study on the bioaccessibility of apple pomace phenolics after in vitro digestion revealed the isomerization and degradation of chlorogenic acids during digestion in the small intestine [139]. In this study, the concentration of chlorogenic acids decreased during the transition from the gastric environment (11−18 mg/100 g) to the intestine (1.7–4.2 mg/100 g); in the intestinal phase of digestion, chlorogenic acid isomers, like cryptochlorogenic acids and neochlorogenic acids, were identified [139]. In addition, Clifford et al. [140] confirmed that chlorogenic acids could be easily hydrolyzed by intestinal esterase to release metabolites (caffeic acids, ferulic acids, and 3′,4′-dimethoxycinnamic acids) which could easily diffuse into epithelial cells of the small intestine. During the intestinal phase, the main apple flavonoids detected in the simulated intestinal juice are flavonols (11.6–18.4 mg/100 g), followed by dihydrochalcones (3.1–4.6 mg/100 g). In the small intestinal juice, high amounts of quercetin glycosides were also detected, with these flavonol derivates being stable in duodenal juice during a 24 h incubation [141,142]. These results could explain the high amount of bioaccessible quercetin glycosides under simulated intestinal conditions, indicating their stability in the intestinal medium. [139,143]. The results on the digestion studies of flaval-3-ol monomers in the intestinal environment showed that (+)-catechin and (−)-epicatechin were readily released from apple pomace and rapidly absorbed in the small intestine [143,144,145]. Tenore et al. [146] studied simulated digestion behaviors of apple phenolics, demonstrating that, in case of procyanidins, the retention was high after intestinal digestion due to their stability under intestinal conditions.

The unabsorbed polyphenols enter the colon, which is populated by microbiota, with one of the most dominant bacterial phyla being *Firmicutes* [147,148]. Kahle et al. [116] reported that only 58% of phenolic compounds from apple were absorbed in the small intestine, with large amounts of unmetabolized phenolics being found in the colon. Numerous studies demonstrated that phenolic compounds are associated with nondigestible cell wall polysaccharides and could accumulate in the colon, interacting further with the gut microbiota [149,150]. Even if the fermentation process could reduce the bioaccesibility of the polyphenols, this process also generates fermented metabolites of phenolics, which may have higher bioactivity than their parent compounds [151]. For example, chlorogenic acid is hydrolyzed under the action of the esterase-rich colonic microbiota, releasing phenolic metabolites, which are converted into soluble forms and absorbed via the colonic epithelium [149,152]. Also, flavonoids and most of the flavan-3-ol oligomers from apples are hydrolyzed by microbial enzymes during the colonic phase, being absorbed at the colon level [153,154].

An in vitro digestion study showed that, after the gastrointestinal digestion, the concentration of bioaccessible phenolic compounds in carrot waste was increased by 13-fold [130]. Chlorogenic acid from carrot waste was the principal compound released in the intestinal phase, representing about 75% of the total bioaccessible compounds [130]. In the soluble colonic fraction, the total phenolic concentration decreased (>75%) compared to the small intestine fraction. Also, flavonols, flavanones, and flavones were not detected in the colonic fraction, probably due to their metabolization by the microorganisms present in the gut [130]. Small amounts of chlorogenic acid were bioaccessible after colonic digestion. Again, the low amounts of phenolic compounds that remained after colonic digestion can be explained by the chemical transformation of the polyphenols under the action of gut microbiota [155].

## 4. The Effects of Bioactive Compounds from Apple, Carrot and Their by-Products on Post-Weaning Piglets: Focus on Intestinal Health

### 4.1. Effect of Bioactive Compounds from Apples, Carrots and Their by-Products on Weaned Piglets’ Performances

Knowing the negative effects of the weaning period on the animal performances in piglets, the data on the attenuation of these effects through diets including apple or carrot wastes were collected and are presented here.

The effects of the diet with apple pomace on weaned piglets’ performances are inconsistent. For example, a diet including apple pomace (3.5%) or polyphenols from apple pomace (400 mg or 800 mg) did not affect the energy intake, feed uptake, average daily gain, or the weight gain in weaned piglets [156,157]. In contrast, Dufourny et al. found that a 4% inclusion of dried apple pomace in the diet of post-weaning piglets improved the average daily gain of piglets and reduced the feed conversion rate and energetic feed efficiency compared to piglets fed a control diet [158]. An increased average daily gain was reported in weaned piglets receiving a 10% fermented apple pomace diet, with no effect on average feed intake, feed/gain ratio or diarrhea rates [159]. These authors explained the promotion of growth performance in monogastric animals through the increase in digestible crude protein in apple pomace after fermentation, which facilitates its digestion and absorption [159,160].

For carrot wastes, the data on their effects on animal performances in piglets are very limited, with the studies being mainly focused on the effects of carotenoids and especially β-carotene. A study carried out by Jugl et al. [161] showed that 10% carrot powder included in the diet of post-weaning piglets did not affect the weight gain and feed intake but reduced the diarrhea incidence compared to control group. The addition of β-carotene to the piglets’ diet (40 or 80 mg/kg body weight, respectively) slightly increased the average daily gain and significantly alleviated the diarrhea incidence compared to the control group [162].

### 4.2. Effect of Bioactive Compounds from Apples, Carrots and Their by-Products on the Intestinal Morphology and on the Intestinal Mechanical Barrier

At weaning, one of the majorly affected organs is the small intestine, with the intestinal architecture being profoundly disturbed via structural changes, which affect the absorption processes [24,25]. The small intestine is the most important site for the absorption of nutrients, with its efficiency being correlated to the measurements of the intestinal surface area; the villus length, crypt depth and the villus length/crypt depth ratio are good indicators of the digestive capacity of the small intestine [163]. A study carried out by Sehm et al. [156] showed a beneficial effect of the inclusion of 3.5% of polyphenol-rich apple pomace in the diet (5.8 mg polyphenols/g DM) on the villus’ height in the jejunum and colon of post-weaning piglets. The effects were pronounced on the Peyer’s patches located in the ileum segment, which were reduced in the apple-pomace-fed group, indicating the immuno-preventive effect of polyphenols from apple pomace in the ileum-associated immune system [156]. The same study reported an increased crypt area in the colon induced by the apple pomace diet, assuming a better nutrient absorption at this level [156]. Also, the diet supplementation with 400 and 800 mg apple polyphenols/kg of body weight ameliorates the jejunum villi height, improving intestinal absorption capacity in weaned piglets [164]. In the duodenum and jejunum of piglets, the supplementation of the diet with chlorogenic acid 1000 mg/kg increased the villus’ height [165]. A diet containing 200 mg/kg apple pectic oligosaccharide increased the villi’s height and reduced the villus’ height/crypt depth in the jejunum of rotavirus-challenged weaned piglets [166]. Morphological measurements of small intestinal segments showed that piglets receiving the 4% whole apple pomace (rich in mono- and disaccharides, fiber and polyphenols) in the diet presented higher duodenal villus length and a higher ileal ratio villus length/crypt depth than piglets fed the control diet [158]. These results demonstrated that the positive effects of apple pomace on intestinal morphology could be attributed not only to their composition in polyphenols, but also to the presence of mono- and disaccharides and of pectic oligosaccharides. Weaning stress could also lead to an impairment of the physical barrier, the disruption of intestinal epithelial tight junctions, followed by an increased intestinal permeability [30,163]. A total of 200 mg of apple pectin oligosaccharides in rotavirus-infected weaned piglets enhanced the expressions of ZO-1, occludin, CLDN 1 and CLDN 3, and the concentrations of mucins MUC1 and MUC2 in the jejunal mucosa of weaned pigs [166], while pectin of apple origin enhanced the expression of *MUC2* and *MUC3* in the jejunum of rats [167]. Apple polyphenols improved the jejunal barrier function by increasing the mRNA levels of *occludin*, *MUC-1*, and *MUC-4* and up-regulating the protein expression of occludin [164]. These results suggested that the bioactive compounds from apple could improve the mucosal–epithelial integrity, intercellular junctions between the epithelial cells, and the mucus layer in weaned piglets. The amelioration of the barrier function by apple polyphenols was also demonstrated in an in vitro study on porcine IPEC-J2 cells through the modulation of tight-junction protein expressions (ZO-1, occludin and claudin-1) [168]. Also, colon Caco-2 cells exposed to crude apple extract and to apple homogenate digested in vitro had an increased expression of CLDN1, CLDN4 and ZO-2 junction proteins [169].

Unlike apples and apple pomace, there are scarce data in the literature attesting to the effect of carrot and carrot by-products on the barrier functions of the intestine. However, the dietary supplementation of β-carotene (diets with 40 mg/kg body weight and 80 mg/kg body weight, respectively, reduced the jejunum villi height and the ratio of the villus’ height/crypt depth compared to weaned piglets [162]. Also, the architecture of the colonic crypts was restored via the addition of β-carotene in the diet [162].

### 4.3. Effect of Bioactive Compounds from Apples, Carrots and Their By-Products on Oxidative Stress and Inflammation at Intestinal Level

At the intestinal level, oxidative stress is a major contributor to tissue destruction due to the imbalance between pro-oxidant and antioxidant responses, and is one of the targets of the nutritional strategies dedicated to weaned piglets. Knowing the well-described antioxidant role of polyphenols, it is expected that apple wastes rich in these biomolecules modulate the oxidative stress in weaned piglets. Indeed, there are studies which demonstrate the antioxidant effects of dietary apple polyphenols in weaned piglets, both at the intestinal level, in the jejunum (increase in the SOD and GPx activities and in total antioxidant capacity TAC) [164] and in serum (increased TAC level and significantly reduced the content of malondialdehyde-MDA) [157]. Also, apple pectic oligosaccharides (200 mg/kg of diet) enhanced TAC and decreased MDA levels in the jejunal mucosa of rotavirus-infected weaned piglets [160]. In other species, for example, in mice, it has been reported that apple polyphenol treatment increased the activities of CAT, GPx and SOD, strengthening the defense against oxidative injury [170]. Lambs fed a 10% fermented apple pomace diet demonstrated a higher antioxidant activity in plasma [171], while Wistar rats fed a diet with 14% apple pomace had an increased SOD activity in red blood cells [172]. These studies demonstrated the potential of apple pomace bioactive compounds in mitigating oxidative stresses at local (intestinal) and systemic (plasma) levels.

The antioxidant role of β-carotene, an important bioactive compound from carrot and its by-products, was demonstrated against oxidative stress and lipid peroxidation [173]. It was found that β-carotene decreased thiobarbituric reactive substances (TBARS, a marker of lipid peroxidation) and increased GSH levels in the colon of mice with DSS-induced ulcerative colitis; also, the same study reported that β-carotene decreased oxidative DNA damage in the colon of mice, substantiating its antioxidant property [173].

Another important focus in the nutrition of weaned piglets is the suppression of intestinal inflammation. Again, one of the main compounds with anti-inflammatory roles is polyphenols, also found in apple waste, which are useful in alleviating intestinal inflammation. Apple polyphenols increased the concentration of serum immunoglobulin A (IgA) in weaning piglets [164]. In contrast, Ao et al. [159] found no effects at the level of plasma immunoglobulins, IgA and IgG in weaned piglets fed a diet with 5% *Saccharomyces cerevisiae*-fermented apple pomace. Apple polyphenols decreased the mRNA levels of *TNF-α*, *IL-1β*, and *IL-8* in the jejunum of piglets after weaning [164]. Also, chlorogenic acid (polyphenol found in many fruits, and in apple) decreased the serum TNF-α, IL-1β, and IL-6 concentrations in weaned pigs [165]. In contrast, a diet with 3.5% apple pomace did not affect the TNF-α, IL-1β, and IL-10 levels in the small intestine of weaned piglets [156]. Different concentrations of apple polysaccharides effectively decreased the IFN-β, TNF-α, and IL-6 levels induced by azoxymethane/DSS in vivo (mice received dietary concentrations of 1.5%, 2.5% and 5% of apple polysaccharides) and by LPS (lipopolysaccharide) in vitro in HT-29 cells treated with 1 mg/mL apple polysaccharides [174]. Also, apple pectin reduced the proinflammatory cytokines IL-1β, IL-8, IL- 6 and IL12 in the ileum and cecum of weaned piglets [105]. Studies performed on an apple polyphenol, luteolin, showed a suppression of the phosphorylation of I*κ*B and the accumulation of NF-κB in the cytoplasm, alleviating the epithelial inflammation in the colon cell line Caco-2 pre-treated with decabromodiphenyl ether (BDE-209) [175]. Also, in a mice model of intestinal inflammation induced by DSS challenge, luteolin suppressed the expression of NF-κB/p65 protein in the colon [176].

Literature data do not provide evidence regarding the anti-inflammatory role of carrot/carrot wastes. However, it was demonstrated that carotenoids play important roles in cellular membrane protection against damage caused by peroxyl radicals, having a scavenging function against reactive oxygen species and anti-inflammatory roles [177]. β-carotene, a natural carotenoid found in many vegetables, including carrot, has potential anti-inflammatory and antioxidant functions, decreasing MDA levels and increasing SOD and GPx activities in serum of piglets [178]. β-carotene (40 and 80 mg/kg body weight) reduced the pro-inflammatory mediators (TNF-α, IL-1β, and IL-6) in serum [162] and restored the mRNA levels of *IL-6* in the jejunum and of *TNF-α* in the colon of piglets after weaning [162]. The β-carotene treatment significantly reduced myeloperoxidase (MPO) activity and the levels of IL-17, IL-6, TNF-a, and COX-2 inflammatory markers in the colon of mice with DSS-induced colitis [173]. In an in vitro model, β-carotene suppressed the production of IL-1β, IL-6, and TNF-α in macrophages treated with LPS- [179]. Treatment with β-carotene suppressed the transcription of *IL-1β*, *IL-6* and *IL-12 p40* cytokines in RAW264 murine macrophages [180] and reduced the production of nitric oxide (NO), prostaglandin (PG)E2, TNF-α, and IL-1β levels in LPS-stimulated rats and in LPS-treated intestinal epithelial cell line (IEC)-6, attenuating LPS-induced inflammation [181].

### 4.4. Effect of Bioactive Compounds from Apples, Carrots and Their by-Products on in-Depth Signalling Pathways

Both inflammation and oxidative stress are regulated by a variety of mechanisms involving multiple signaling pathways.

NF-κB is the main intracellular pathway involved in the inflammatory response, along with other classical inflammatory pathways: MAPKs, IL-6/JAK/STAT3 and PI3K [182]. Under physiological circumstances, NF-κB is sequestered in the cytoplasm by the IKB/IKK enzymatic complex [183]. When the NF-κB is released, the activated form is translocated into the nucleus and increases the transcription of distinct genes involved in the synthesis of pro-inflammatory cytokines [184,185]. Apple polyphenols reduced the NF-κB activation in both in vivo studies on rats [186] and mice [170] and in vitro studies on human and mice cells [187,188,189]. In weaned piglets, a diet with 5% pectin from apple fruit downregulated the expression of *NF-κB* gene in the ileum and cecum segments [105]; also, polyphenols extracted from apple reduced the expression of *NF-κB* in the jejunum of weaned piglets [164].

The link between oxidative stress and inflammation is confirmed by the crosstalk between their important regulators, Nfr2 and NF-κB [190]. It was demonstrated that a deficient Nrf2 activation induced NF-κB expression, leading to an intense transcription of genes coding for inflammatory factors. In turn, activated NF-κB can regulate the transcription and activity of Nrf2 at the translational level [190].

The phytochemicals from apple pomace, particularly polyphenols, act as Nfr2 inducers, activating the Nrf2 signaling pathway [191]. The induction of Nrf2 signaling by apple polyphenols was demonstrated via both in vitro and in vivo studies. The molecular mechanisms of thinned apple polyphenol (TAP) fractions consisted of 24% of polyphenols, shown in cell models (with gene reporters for Nfr2) dose-dependent antioxidant activities [186]. Nrf2 is an important mediator of endogenous mechanisms of defense [192]. Similar to NF-κB, Nrf2 is sequestered by Kelch-like ECH-associated protein 1 (Keap1) into the cytoplasm and is degraded in proteasome via ubiquitination [193]. In response to oxidative stress, the Nrf2-Keap1 complex is disrupted, and the activated Nrf2 is released and translocated into the nucleus, where it binds to antioxidant response elements (ARE) [194,195], up-regulating the protective antioxidant enzymes and the heme-oxygenase HO-1 gene expressions [196]. Treatment with apple polyphenol extracts enhanced Nrf2 nuclear translocation, as well as the activation of Nrf2 and of regulatory factors such as HO-1 [197]. Also, polyphenols from different apple sources (apple juice, smoothies, pomace) activate Nrf2 in in vivo studies on the colon and liver of rats and piglets, respectively [168,198,199,200]. A recent study on weaning piglets supplemented with apple polyphenols (400 mg/kg and 800 mg/kg) confirmed an Nrf2 activation in the jejunum and intestinal mucosa, and, through an in vitro study using IPEC-J2 cells, Huang et al. demonstrated that the Nrf2/Keap1 pathway modulates the antioxidant effects of apple polyphenols at the intestinal level [168]. Another in vivo study showed that apple polyphenols up-regulated the protein expressions of NQO1, HO-1 and Nrf2 and down-regulated the protein expression of Keap1 in the jejunum of piglets after weaning [164].

The mechanism through which carotenoids interfere with oxidative stress and inflammatory responses are also interactions with cellular signaling cascades, such as the NF-κB, MAPK, Nrf2 pathways [177]. In a study by Zhang et al. [192], β-carotene at 5 to 50 μM concentrations up-regulated the expression of Nrf2 in K562 leukemia cells. In rats, β-carotene significantly attenuated NF-κB, Akt, JAK2/STAT3 (Janus kinase 2/signal transducers and activators of transcription 3), and c-Jun N-terminal kinase (JNK)/p38 mitogen-activated protein kinase (MAPK) signaling proteins activated by LPS stimulation [181]. Also, a similar modulation of β-carotene on LPS-treated intestinal cell line IEC-6 cells was reported [181]. In weaned piglets’ jejunum, β-carotene decreased the activation of JNK/p38 MAPK [178]

### 4.5. Effect of Bioactive Compounds from Apples, Carrots and Their by-Products on the Microbiota Composition

Another component affected by weaning transition in piglets is the gut microbiota. There is increasing evidence showing that dietary bioactive compounds can modulate the gut microbiome, increasing the abundance of beneficial bacteria or decreasing the levels of harmful microbial species in the gut microbiota [201]. In turn, the gut microbiota can metabolize these bioactive compounds into low-molecular-weight metabolites, which can modulate the regulatory metabolism network [201]. Of bioactive compounds from apple residues, polyphenols and their interaction with gut microbiota were the most studied in both in vivo (weaned piglets) and in vitro (fermentation) studies.

In vivo works have shown that apple wastes can modulate gut microbiota in piglets after weaning. Briefly, the richness of the microbiota in the feces is increased in piglets fed a 4% apple pomace diet, demonstrating the microbiota’s stability and resilience after the weaning-stress-related disturbance [158,202]. It was demonstrated that both an apple pomace diet (4%) and a fermented apple pomace diet (10%) increased the abundance of beneficial bacteria *Lactobacillaceae* in the feces of piglets after weaning [158,159]. Also, *Lactobacillaceae* and *Faecalibacterium* were increased in the feces of piglets fed a 5% apple pectin diet [105]. These bacterial species are associated with intestinal health [203], with other studies demonstrating the positive effects of apple pectin on these genera in both in vitro fermentation models [204] and in humans fed pectin from apple [205]. A bacterium which seemed to be specifically modulated by the apple bioactive compounds is *Catenibacterium*, which was increased in the feces and in the proximal colon microbiota as a result of dietary apple pomace supplementation of post-weaning piglets [158]. Others have also shown that a diet containing garlic acid and apple pomace (3%:3%) increases the abundance of *Catenibacterium* in enterotoxigenic *Escherichia coli* (ETEC)-challenged weaned piglets [206]. This genus is a Gram-positive bacteria that produces lactic, acetic, butyric and iso-butyric acids [207] and has previously been reported to be exclusively present in pigs fed dietary fiber [208,209]. *Catenibacterium* was previously reported to be reduced in intestinal inflammation pathologies and was linked to the abundances of *Prevotella* [210]. Indeed, in weaned piglets fed 5% apple pectin or 4% apple pomace diets, an increased level of *Prevotella* was found [105,158]; *Prevotella* has been suggested to have anti-inflammatory properties in animal gut [211] and to positively modulate post-weaning diarrhea in piglets [212].

A study carried out by Dufourny et al. showed that a 4% apple pomace diet reduced the *Clostridiaceae* abundances in the feces of piglets after weaning [158]. A reduction in the relative abundances of the *Clostridium sensu stricto 1*, *Terrisporobacter*, and *Ruminococcus* is reported in piglets fed a 10% fermented apple pomace diet [159]. Data from human studies showed that an increased level of *Clostridium* species causes inflammatory diseases [213], and a meta-analysis reported that polyphenols derived from different foods suppress the abundance of pathogenic *Clostridium* species in the human gut microbiota [214].

Data on the effects of carrot wastes on gut microbiota are very limited, with the studies being focused only on the effects of β-carotene. An analysis on the composition of fecal microbiota in post-weaning piglets fed a diet supplemented with β-carotene (40 mg/kg body weight or 80 mg/kg of body weight) showed an increase in the abundance of species from the phyla *Firmicutes* and the genera *p-75-a5*, and *Parabacteroides* [162], and a reduction in the species from phyla *Bacteroidetes* and the genus *Prevotella*, and *Blautia* was found [162]. The authors performed a correlation analysis showing that *Prevotella* and *Blautia* were positively correlated with the concentration levels of IL-1β, IL-6, and TNF-α pro-inflammatory cytokines in serum of piglets, while *Parabacteroides* and *Synergistes* were negatively correlated [162]. β-carotene supplementation also decreased the abundance of harmful bacteria like *Dialister* and *Enterobacter* [162,215]. In addition, the effects of β-carotene on the composition of the intestinal microbiota seemed to be dose-specific, as low and medium doses increased the *Bifidobacterium* abundance, while high doses increased the abundance of *Lactobacillus* [215].

## 5. Conclusions

This review gave an overview of the effects of bioactive compounds from apple and carrot and their by-products on the main aspects affected by the weaning transition: growth performance, diarrhea incidence, intestinal morphology, oxidative stress and inflammation markers, their associated in-depth signaling pathways, and the intestinal microbiota. It was highlighted that these compounds (especially polyphenols and pectin) could enhance the intestinal health in piglets after weaning, reducing the oxidant and inflammatory processes and positively modulating the microbiota. There are more studies investigating the effects of apple and apple wastes and their bioactive compounds on weaned piglets, although data on the same effects of carrots and their wastes are very limited. The scientific information presented in this review indicated that apple and apple wastes could be used in the nutrition of weaning piglets, whereas for carrot and its by-products, more studies should be developed. In the context of the development of a circular economy emphasized by the European Union, these by-products are an inexpensive source of beneficial bioactive compounds and can be used in the feeding of weaning piglets as valuable replacers of in-feed antibiotics and ZnO.

## Figures and Tables

**Table 1:** The basal composition of apple fruit and apple pomace.

Components *	Apple	Apple Pomace
Total protein	0.19–3.3% [85,86]	2.4–11% [77]
Crude fiber	2.00–2.97% [87]	11.6–44.5% [77]
Neutral detergent fiber (NDF)	0.9–1.6% [88]	42.07% [89]
Acid detergent fiber (ADF)	1.0–1.5% [88]	34.27% [89]
Cellulose	0.43% [77]	12.0–23.2% [77]
Lignin	15.3% [77]	6.4–19.0% [77]
Hemicellulose	19.2% [77]	5.0–6.2% [77]
Pectin	1.0–1.5% [86]	3.5–18% [77,90]
Sugars	10.4–12.34% [87,91]	54.4% [82,83]
Fructose	4.4–6.9% [92]	14–35% [82,83,85]
Glucose	1.4–3.4% [92]	2.5–13% [82,83,85,93]
Sucrose	1.7–7.4% [85]	3.4–24% [85]
Crude fat	0.1–1.9% [85,87]	2.7% [94]
Ash	0.19–1.7% [85,87]	1.48–4.00% [89,93]
Macroelements		
K	0.09–1.18% [86,87]	0.449% [89]
P	0.13–0.84% [86,87]	0.070–0.149% [77,80,84,89]
Ca	2.48–7.8% [87,91]	0.06–0.15% [77,80,84,89]
Mg	0.27–3.46% [86]	0.02–0.45% [77,80,84,89]
Microelements		
Fe	0.15–0.28% [87,91]	31.8–38.3% [77,80,84]
Mn	0.04–0.06% [87]	8.75% [89]
Zn	0.006–0.02% [86]	6.9% [89]
Cu	0.03–0.04% [87]	1.36% [89]

* The composition is expressed as g/100 g dry matter.

**Table 2:** The basal composition of whole carrot and carrot pomace.

Components *	Carrot	Carrot Pomace
Crude protein	0.7–0.9% [103]	6.21–12.87% [99,100]
Crude fibers	1.2–2.4% [103]	8.8–28% [89,99,100]
Neutral detergent fibers (NDF)	- **	12.07% [89]
Acid detergent fibers (ADF)	- **	11.87% [89]
Cellulose	35–48% [103]	- **
Lignin	15.2% [103]	- **
Hemicellulose	13.0% [103]	- **
Sugars	2.71–5.6% [103]	64.3% [101]
Crude fat	0.2–0.5% [103]	1.23–2.72% [99,100]
Ash	1.1% [103]	6.18–7.67% [99,102]
Macroelements		
K	0.24% [103]	0.27% [89]
P	0.25% [103]	0.39% [89]
Ca	0.34% [103]	0.34% [89]
Mg	0.9% [103]	0.12% [89]
Microelements		
Fe	0.4% [103]	11.6–22.3% [89,99]
Mn	0.2–0.8% [104]	13.1% [89]
Zn	0.2% [103]	28.4% [89]
Cu	0.02% [103]	4.24% [89]

* The composition is expressed as g/100 g dry matter. ** Data not found.

**Table 3:** The composition in bioactive compounds of apple, carrot and their pomaces.

Bioactive Compounds *	**Apple**	**Apple Pomace**
Total polyphenols	66.2–434.4% [93,108]	262–856% [93]
Total flavonoids	57–338.6% [108]	94.3% [109]
Total anthocyanins	0.19–2.30% [108]	2.11% [89]
Vitamin E	0.14–0.25% [87]	22.4% [89]
Vitamin C	4.60–77% [87,91]	5.5% [93]
Bioactive Compounds *	**Carrot**	**Carrot Pomace**
Total polyphenols	15.9–25.9% [103]	13.8% [110]
Sum of anthocyanins	1.75% [103]	4.32% [110]
Vitamin E	19.1–70.3% [104]	41.5% [110]
Vitamin C	1.0–4% [103]	30–70% [110]
Carotenoids	5.33% [103]	3.32–15.35% [110]
Lutein	1.9% [104]	0.023–1.61% [110]
Beta-carotene	1.7% [104]	3.26–13.44% [110]
Astaxanthin	- **	0.0147% [110]

* The composition is expressed as g/100 g. ** Data not found.

## Data Availability

Not applicable.
